# A Substitute for Mercury in Syphilitic Diseases

**Published:** 1852-03

**Authors:** 


*A Substitute for Mercury in Syphilitic Diseases.*—M. E. Robin has read a paper before the Academy of Sciences of Paris, with the following title:—"On Certain new Agents calculated as Substitutes for Mercury when used as an Anti-syphilitic Remedy." In former papers, M. Robin has maintained these propositions:—"Mercurial preparations do not act in a peculiar manner when administered in Syphilitic diseases; they merely combine with the virus and change it into a new or inert compound. Now there are a great many substances which form analogous combinations with organized matter, which substances probably have, like mercury, anti-syphilitic virtues; and it will be found that the agents of this class, which have thus been successfully employed, belong to the antiseptic division of remedies which act by combining with the noxious principles. In this manner we can understand whence arise the anti-syphilitic properties of arsenical, gold, silver steel, and antimonial preparations. Hence arises the likelihood of success, if attempts be made to use such organic substances as the bichromate of potash, or sesquichloride of iron, instead of mercurials."

M. Robin induced Dr. Vicenti, of Paris, to try a few experiments with the bichromate of potash to combat syphilis; the salt was employed in three cases with much success, and of these, one was marked by very severe secondary symptoms. Fifteen grains of bichromate were divided into eighty pills, with extract of gentian. One of these was taken night and morning. They agreed pretty well with the stomach, though some opium was necessary to prevent nausea and vomiting. The patient took 240 pills in the space of about three months, and was then quite well of a very intense attack of iritis, accompanied by other syphilitic symptoms, which had almost blinded him. The patient had had an indurate chancre, and had never taken any mercury.—*Ibid.*

